# Fuels for Thought!

**DOI:** 10.3390/ijms10073235

**Published:** 2009-07-15

**Authors:** Clifford Louime

**Affiliations:** College of Engineering Sciences, Technology and Agriculture, Florida A&M University, Tallahassee, Florida, USA; E-mail: Clifford.Louime@famu.edu

When it comes to the marketing of the bioenergy brand, one of the catchiest slogans out these days is “25 by ‘25”. Adopted and supported by industries, academia and government agencies alike, this organization simply aims to supply 25 percent of our energy from renewable resources by the year 2025. By focusing its future efforts on wind, solar and biomass resources, the “25 by 25” initiative is expected to create new jobs, develop novel technologies, help mitigate the effects of global warming and reduce our dependence on fossil fuels.

Now one should ask the question: “*Is that feasible*?” In a statement issued recently, the U.S. administration acknowledged biofuels as a much needed alternative to foreign oil. Such government support of biofuels is a first step in the path forward for the next phase in bioenergy development in the world. In the wake of recent hikes in gas prices and commodities such as corn and soybean, bioenergy is becoming an attractive option for consumers who are feeling the financial pinch of these turbulent times. There is a renewed sense of urgency shared by producers and consumers alike and a clear vision needs to be articulated for the bioenergy industry to really take flight.

Investments in billions of dollars are currently being made in the bioenergy arena. The United States Department of Energy alone invested up to $375 million in three new Bioenergy Research Centers. The main objective of these centers is to increase efficiency and diversification of clean energy sources by accelerating basic research and development in the area of cellulosic ethanol and other biofuels. These centers have set a more ambitious goal than the “25 by 25”, namely the “Twenty in Ten” initiative, which seeks to reduce U.S. gasoline consumption by 20% within 10 years.

On the other side of the Atlantic, the European Commission is addressing the issue of Bioenergy from a similar perspective. In 1995 the Commission established the European Bioenergy Networks (EBN) to promote renewable energy utilization in Europe. The major aim of the EBN is to share knowledge and experience, spread of information, transfer of knowledge and know-how in the bioenergy sector. In addition, the EBN has set out to define proper strategies to overcome barriers limiting the use of biomass at all levels.

On the African continent, several strategies are now being set out to turn Africa into a biofuel superpower. In 2006, several African countries joined forces together to form the PANPP “*Pays Africains Non*-*Producteurs de Pétrole* - non-oil producing African countries” - a “Green OPEC” of sorts.” Their goal is to share the burden of the rising energy costs affecting their already lagging economies. Concurrently, they created a fund for the development of a pan-African biofuels industry. Independent analysts, however, while agreeing on Africa's potential, also pointed to a large number of challenges. Basically, by taking on biofuels, Africa is looking at a whole new host of opportunities and challenges that they will have to face.

A similar picture can be drawn for Southeast Asia and Australia. In all, in order to serve as a weapon in the fight for energy independence, an improved economy with new jobs and a cleaner environment, a new bioenergy industry around the world will need to address the following 10 (ten) points: 1) Who are going to be the winners and losers in this new endeavor? 2) The “Food Vs Fuel” debate – How will growing feedstock for biofuels impact food supplies and food security worldwide? 3) Will more intensive land use draw down our already limited water supply? 4) Are we heading to monoculture production - knowing its possible implications? 5) Are we growing the next invasives or are we moving to a new state of equilibrium in our ecosystems? 6) How will the industry be regulated and how sustainability be ensured? 7) These new business ventures, do they involve local entrepreneurship or outside investors? 8) Can we make bioenergy relatively “Carbon neutral” – also is carbon neutrality necessary – Is it the goal or should we look to develop bioenergy with a carbon deficit in order to combat global climate change? 9) How will issues of environmental equity and justice be addressed? 10) Are there any other social implications involved – such as the current land clearing triggering folks’ displacement in Brazil? These are not simple questions, nor are there simple answers. But by addressing these issues, we may be able to circumvent the slough of unintended consequences that has bogged down the industry for years.

Finally, as we are all aware of, any new endeavors bring pain and setbacks. Most of us agree that it will take an initiative that rivals the “Manhattan Project” both in scale and ambition, being pushed by governments the world over, in order to accomplish the goals set by all these bioenergy centers. Scientific and technological innovations are required in order to fully tap biomass as a major fuel source. Biofuels is just one of many paths towards energy independence. Other sources of renewables deserve equal attention. We hope that a major breakthrough in solar, wind or biomass science and technology could lead to the world getting half of its energy from renewable sources within the next 10 years. After centuries of reliance on fossil fuels to move people and power economies around the world– change is upon us!

So… Let’s get the ball rolling! But let’s make sure we exercise sound and reasonable environmental restraints and judgments.

